# Gingerenone A Induces Antiproliferation and Senescence of Breast Cancer Cells

**DOI:** 10.3390/antiox11030587

**Published:** 2022-03-19

**Authors:** Tzu-Jung Yu, Jen-Yang Tang, Jun-Ping Shiau, Ming-Feng Hou, Chia-Hung Yen, Fu Ou-Yang, Chung-Yi Chen, Hsueh-Wei Chang

**Affiliations:** 1 Graduate Institute of Natural Products, Kaohsiung Medical University, Kaohsiung 80708, Taiwan; u109831002@kmu.edu.tw (T.-J.Y.); chyen@kmu.edu.tw (C.-H.Y.); 2 School of Post-Baccalaureate Medicine, Kaohsiung Medical University, Kaohsiung 80708, Taiwan; reyata@kmu.edu.tw; 3 Department of Radiation Oncology, Kaohsiung Medical University Hospital, Kaohsiung 80708, Taiwan; 4 Department of Surgery, Kaohsiung Medical University Hospital, Kaohsiung 80708, Taiwan; drshiaoclinic@gmail.com (J.-P.S.); mifeho@kmu.edu.tw (M.-F.H.); swfuon@kmu.edu.tw (F.O.-Y.); 5 Division of Breast Surgery, Department of Surgery, Kaohsiung Medical University Hospital, Kaohsiung 80708, Taiwan; 6 Department of Biomedical Science and Environmental Biology, College of Life Science, Kaohsiung Medical University, Kaohsiung 80708, Taiwan; 7 Department of Nutrition and Health Sciences, School of Medical and Health Sciences, Fooyin University, Kaohsiung 83102, Taiwan; 8 Center for Cancer Research, Kaohsiung Medical University, Kaohsiung 80708, Taiwan; 9 Institute of Medical Science and Technology, National Sun Yat-sen University, Kaohsiung 80424, Taiwan

**Keywords:** ginger, oxidative stress, senescence, DNA damage, breast cancer

## Abstract

Ginger is a popular spice and consists of several bioactive antioxidant compounds. Gingerenone A (Gin A), a novel compound isolated from *Zingiber officinale*, is rarely investigated for its anti-breast-cancer properties. Some ginger extracts have been reported to initiate senescence, an anticancer strategy. However, the anticancer effects of Gin A on breast cancer cells remain unclear. The present study aims to assess the modulating impact of Gin A acting on proliferation and senescence to breast cancer cells. Gin A diminished the cellular ATP content and decreased the cell viability of the MTS assay in several breast cancer cell lines. It also showed a delayed G2/M response to breast cancer cells (MCF7 and MDA-MB-231). *N*-acetylcysteine (NAC), an oxidative stress inhibitor, can revert these responses of antiproliferation and G2/M delay. The oxidative stress and senescence responses of Gin A were further validated by increasing reactive oxygen species, mitochondrial superoxide, and β-galactosidase activity, which were reverted by NAC. Gin A also upregulated senescence-associated gene expressions. In addition to oxidative stress, Gin A also induced DNA damage responses by increasing γH2AX level and foci and generating 8-hydroxyl-2′-deoxyguanosine in breast cancer cells, which were reverted by NAC. Therefore, Gin A promotes antiproliferation and senescence of breast cancer cells induced by oxidative stress.

## 1. Introduction

Breast cancer is the most commonly diagnosed cancer type and the leading cause of cancer death in women, according to 2020 global cancer statistics [1]. Breast cancer accounts for 11.7% of all cancer cases, and several valuable markers have been advocated, such as estrogen receptor (ER), progesterone receptor (PR), human epidermal growth factor receptor 2 (HER2), and Ki67 [2]. For the purposes of therapy, breast cancer is often classified into four subtypes: luminal type, pure HER2, triple-positive, and triple-negative. The triple-negative breast cancer (TNBC) is the most challenging category to treat and can be classified into more subgroups according to gene expression. The most commonly applied classification methods are Lehmann, with six subtypes, and Burstein, with four subtypes (luminal-androgen receptor expressing, basal, claudin-low, and claudin-high) [3,4]. Therefore, effective drug discovery for breast cancer therapy should cure several heterogeneous breast cancer subtypes.

Ginger is known to be “generally recognized as safe” (GRAS) by the Food and Drug Administration (FDA) of the USA [5]. Ginger is popularly used as a spice. Ginger has been used to treat several symptoms such as nausea, vomiting, abdominal pain, and muscle discomfort [6,7]. Extracts from rhizomes of ginger contain several bioactive compounds, such as gingerols, shogaols, gingediols, zingerone, dehydrozingerone, gingerinone, and diarylheptanoids [8,9,10]. These ginger-derived bioactive compounds exhibit anti-inflammatory, antioxidant, and anticancer effects [8,9,10].

In our previous work, the new compound, namely gingerenone A (Gin A), was isolated from *Zingiber officinale* (*Z. officinale*) in 2015 [11]. It demonstrated anticancer, apoptosis, and oxidative stress-inducing effects of Gin A in liver cancer Sk-Hep-1 cells [11]. Other biological functions of Gin A have rarely been investigated. Moreover, the anticancer effects of Gin A for different cancer types are rare, and more detailed mechanisms have not been fully reported.

Inducting senescence is an effective anticancer strategy [12,13]. For example, doxorubicin, etoposide, camptothecin, and cisplatin can promote senescence in several cancer cells [12]. Oxidative stress can also trigger senescence [14]. Although Gin A can improve oxidative stress in liver cancer cells [11], the possible oxidative-stress-inducing senescence and its impact in terms of proliferation to breast cancer cells remain unclear. To answer these questions, this study aimed to evaluate the antiproliferation function and assess the senescence response of Gin A to breast cancer cells.

## 2. Materials and Methods

### 2.1. Plant Material, Extraction, and Isolation

The extraction and isolation steps were performed as previously described [11]. In brief, the rhizomes of the ginger *Z. officinale*, which was identified by Dr. Fu-Yuan Lu (Department of Forestry and Natural Resources College of Agriculture, National Chiayi University), were harvested from Kaohsiung and deposited at the Department of Medical Technology, School of Medical and Health Sciences, Fooyin University, Kaohsiung, Taiwan. The rhizomes (3.1 kg) of *Z. officinale* were soaked repeatedly with MeOH for 24–48 h at RT. After several procedures, the Gin A (1.3 g) was purified. Gin A was dissolved in dimethyl sulfoxide (DMSO) (Sigma-Aldrich; St. Louis, MO, USA) for cell experiments.

### 2.2. Gin A Chemical Profile

Gin A, brown oil. UV (λ_max_, nm): 230, 280. IR (ν_max_, cm^−1^): 3430, 1700, 1615, 1515, 1035, 975. ^1^H NMR (400 MHz, CDCl_3_, δ, ppm, J/Hz): 2.48 (2H, q, J = 7.2, H-6), 2.70 (2H, t, J = 7.2, H-7), 2.84 (4H, m, H-1, 2), 3.86 (6H, s, 3′, 3″-OCH_3_), 5.50 (1H, s, OH), 5.51 (1H, s, OH), 6.11 (1H, d, J = 16.0, H-4), 6.65 (1H, d, J = 2.0, H-2′), 6.66 (1H, dd, J = 8.0, 2.0, H-6′), 6.69 (1H, d, J = 2.0, H-2″), 6.70 (1H, dd, J = 8.0, 2.0, H-6″), 6.82 (1H, d, J = 8.0, H-5′), 6.83 (1H, d, J = 8.0, H-5″) [11].

### 2.3. Cell Culture and Chemicals

HER2+, luminal A, and Claudin-low subtypes [15,16] of breast cancer cells (SKBR3, MCF7, and MDA-MB-231) were collected from American Tissue Culture Collection (ATCC, Manassas, VA, USA). A normal human breast cell line (M10) was collected from the BCRC Cell Bank (HsinChu, Taiwan) [17]. Cells were maintained in a humidified atmosphere of 5% CO_2_ at 37 °C, culturing with a 3:2 mixture of two kinds of culture medium (Dulbecco’s Modified Eagle Medium (DMEM)/F12) (breast cancer cells) or alpha medium (normal breast cells) containing 10% bovine serum (Gibco, Grand Island, NY) and common antibiotics. *N*-acetylcysteine (NAC) (Sigma-Aldrich, St. Louis, MO, USA) [18,19,20,21] was pretreated (10 mM, 1 h), and Gin A was post-treated with cells to evaluate the contribution of oxidative stress in each experiment.

### 2.4. Cell Viability

Viability was assessed using an ATP kit (PerkinElmer Life Sciences, Boston, MA, USA) [22], MTS cell proliferation assay [23], and trypan blue reagent [24] based on the user manual’s instruction.

### 2.5. Cell Cycle Detection

To stain DNA, 7-Aminoactinomycin D (7AAD) (Biotium; Hayward, CA, USA) (1 μg/mL, 30 min, 37 °C) was used in order to determine cell cycle phases [23]. The 7AAD-stained cells were resuspended in PBS to perform flow cytometer (Guava easyCyte; Luminex, TX, USA). Cell cycle phases were analyzed by FlowJo software version 10 (Becton-Dickinson; Franklin Lakes, NJ, USA).

### 2.6. ROS Detection

ROS-positive populations were detected by staining with 2′,7′-dichlorodihydrofluorescein diacetate (H_2_DCF-DA) (Sigma-Aldrich, St. Louis, MO, USA) (10 μM, 30 min, 37 °C) as mentioned in [23]. The ROS intensity was assessed by Accuri C6 flow cytometer (Becton-Dickinson).

### 2.7. Mitochondrial Superoxide (MitoSOX) Detection by Flow Cytometry

MitoSOX-positive populations were detected by staining with MitoSOX™ Red (Thermo Fisher Scientific, Waltham, MA, USA) (50 nM, 37 °C, 30 min), as previously described [25]. The MitoSOX intensity was assessed by Accuri C6 flow cytometer.

### 2.8. Senescence Detection

Senescence was determined by monitoring the expression of β-galactosidase using flow cytometry and fluorescence microscopy. For flow cytometry, senescent cells were mixed using a Senescence Detection Kit (Fluorometric) (ab228562; Abcam) [26] with the requirements of 37 °C and 60 min, measured by flow cytometer (Guava easyCyte), and analyzed by FlowJo software.

For fluorescence monitoring, cells were fixed with 4% paraformaldehyde for 10 min. After washing, cells were incubated with senescence fluorescent dye (Abcam) at 1:1000 and bisBenzimide H 33342 trihydrochloride (Sigma-Aldrich) 1:1000 for 2 h at 37 °C without CO_2_. Cells were washed three times with PBS and mounted with Dako Fluorescence Mounting Medium (Invitrogen, Grand Island, NY, USA). Slides were photographed using a DMi8 microscope (Leica Microsystems, Wetzlar, Germany).

### 2.9. Senescence Gene Expression Detection

cDNA was prepared from drug-treated cells for quantitative RT-PCR (qRT-PCR) [27]. Senescence-related genes [28,29], including endothelin 1 (*EDN1*), ankyrin repeat domain 1 (*ANKRD1*), cyclin-dependent kinase inhibitor 1A (*CDKN1A*), serpin family E member 1 (*SERPINE1*), and transgelin (*TAGLN*) were selected to perform touch-down PCR [27], accompanied by the use of glyceraldehyde 3-phosphate dehydrogenase (*GAPDH*) as an internal control. The gene expression (log_2_) was calculated using the 2^−ΔΔCt^ method [30]. The primer information for senescence-related genes (*EDN1* NM_001955.5, *ANKRD1* NM_014391.3, *CDKN1A* NM_000389.5, *SERPINE1* NM_000602.5, and *TAGLN* NM_001001522.2), and GAPDH (NM_002046.7) [31,32] is shown in Table 1.

### 2.10. DNA Damage Detection

DNA damage detection was measured by γH2AX-based flow cytometry and fluorescence microscopy. For flow cytometry [33,34], γH2AX-positive populations were detected for antibody reactions, i.e., γH2AX antibody 1:500 (Santa Cruz, CA, USA) and Alexa Fluor 488-secondary antibody 1:10,000 (Cell Signaling Technology, Danvers, MA, USA). In addition, cells were counterstained with 7AAD (5 μg/mL, 30 min). Finally, cells were analyzed using an Accuri C6 flow cytometer.

For γH2AX foci detection [33], cells were processed with 4% paraformaldehyde fixation for 10 min. Then, cells were permeabilized with 0.1% Triton X-100 for 5 min and processed with 1% BSA blocking for 1 h. Subsequently, γH2AX foci positive cells were detected by antibody reaction, i.e., γH2AX antibody 1:400 and Alexa Fluor 488-secondary antibody 1:500. In addition, cells were counterstained with bisBenzimide H 33342 trihydrochloride (Sigma-Aldrich) 1:1000, combined with Dako Fluorescence Mounting Medium (Invitrogen, Grand Island, NY, USA), and, finally, obs, erved by Leica DMi8 microscope (Wetzlar, Germany).

### 2.11. 8-Hydroxyl-2′-Deoxyguanosine (8-OHdG) Detection

The marker 8-OHdG is used for detecting oxidative DNA damage [35,36], and it can be measured with 8-OHdG-based flow cytometry [33]. Populations positive for 8-OHdG were detected using antibody reaction, i.e., antibody 8-OHdG (E-8) FITC 1:10,000 (4 °C, 1 h). Subsequently, cells were analyzed by Accuri C6 flow cytometer.

### 2.12. Statistics

ANOVA after Tukey’s HSD Post hoc Tests (JMP^®^12 software, SAS Institute Inc., Cary, NC, USA) was applied for statistical analysis to assess multicomparison. Different treatments were labeled with connecting letters. When the connecting letters were overlapping, the difference between them was non-significant. In contrast, their difference was significant when the connecting letters were overlapping.

## 3. Results

### 3.1. Gin A Diminished Proliferation of Breast Cancer Cells

**Figure 1:**
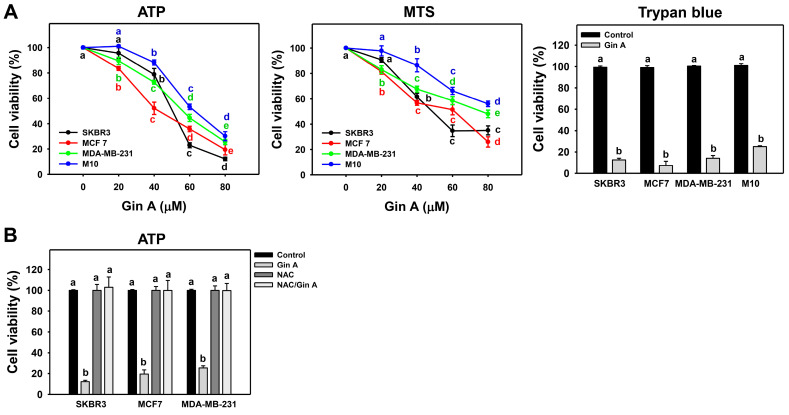
Impact of Gin A on cell viability of breast cancer cells. (**A**) Cell viability. Cells were treated with 0 (0.1% DMSO; control), 20, 40, 60, and 80 μM Gin A for 48 h. Subsequently, they were subjected to ATP, MTS, and trypan blue (80 μM) assays to determine cell viability. (**B**) NAC reverted effects on antiproliferation. Cells were pretreated with NAC (10 mM) for 1 h and then post-treated with Gin A (control and 80 μM) for 48 h, namely NAC/Gin A. Data, means ± SDs (*n* = 3). When the connecting letters are overlapping, it means their difference was significant for multiple comparisons (*p* < 0.05 to 0.0001). For example, control, NAC, and NAC/Gin A showing *a* differ non-significantly. These three treatments marked with *a* differ significantly from Gin A marked with *b*.

Gin A (0, 20, 40, 60, and 80 μM) treatment for 48 h inhibited cell viability (ATP and MTS assays) of several kinds of cell lines of breast cancer in a dose-dependent manner (Figure 1A). In 48 h TP assay, Gin A exhibited IC_50_ values of 50.41, 42.67, and 56.29 μM in breast cancer cells (SKBR3, MCF 7, and MDA-MB-231, respectively). In 48 h MTS assay, Gin A exhibited IC_50_ values of 48.91, 61.40, and 76.12 μM in breast cancer cells (SKBR3, MCF 7, and MDA-MB-231, respectively). Trypan blue analysis shows similar cytotoxic results. Normal breast cells (M10) show higher cell viability than breast cancer cells. By adding the ROS scavenger NAC, we assessed the impact of oxidative stress in antiproliferation of Gin A on breast cancer cells. After NAC pretreatment, Gin A-induced antiproliferation effects were suppressed, and they were recovered to 100% viability-like control (Figure 1B).

### 3.2. Gin A Delayed G2/M Progression of Breast Cancer Cells

**Figure 2:**
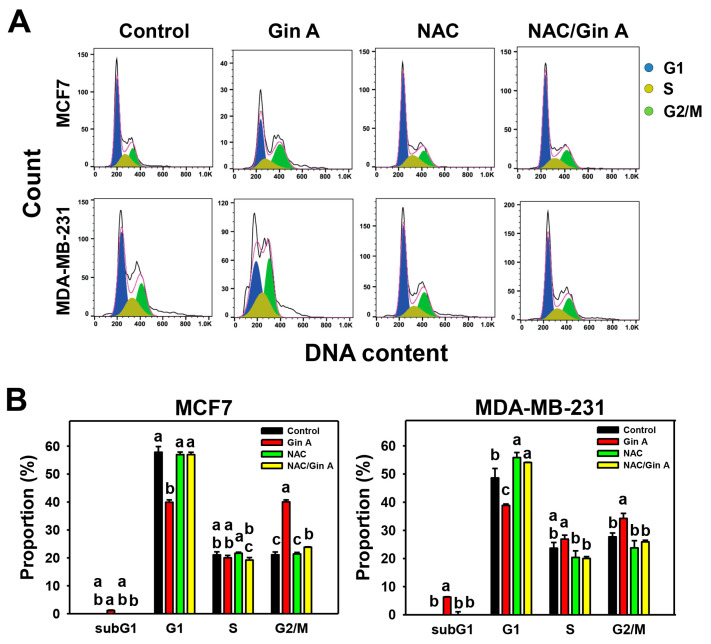
Impact of Gin A on cell cycle of breast cancer cells. (**A**,**B**) NAC reverted the effects on cell cycle disturbance. Cells were pretreated with NAC (10 mM) for 1 h and then post-treated with Gin A (control and 80 μM) for 48 h, namely NAC/Gin A. Data, means ± SDs (*n* = 3). When the connecting letters are overlapping, their difference was significant for multiple comparisons (*p* < 0.05 to 0.0001). For example, the G1 phase for MCF7 cells (**B**), control, -NAC, and NAC/Gin A showing *a* differ non-significantly because they overlap with the same letter. These three treatments marked with *a* differ significantly from Gin A marked with *b.* For the example of the G2/M phase for MCF7 cells (**B**), Gin A, control, and NAC/Gin A showing *a*, *c*, and *b* differ significantly because they do not overlap with the same letter.

The impact of Gin A on the cell cycle of breast cancer cells (MCF7 and MDA-MB-231) was assessed using flow cytometric analysis (Figure 2A). Gin A (80 μM) treatment for 48 h showed a minor increase in subG1, a moderate decrease in G1, and an increase in G2/M populations compared to control (Figure 2B).

By adding the ROS scavenger NAC, we assessed the impact of oxidative stress on cell cycle regulation of Gin A in breast cancer cells. After NAC pretreatment, Gin A-induced subG1 accumulation, G1 decrement, and G2/M increment were reverted (Figure 2B).

### 3.3. Gin A Provoked ROS Increment in Breast Cancer Cells

**Figure 3:**
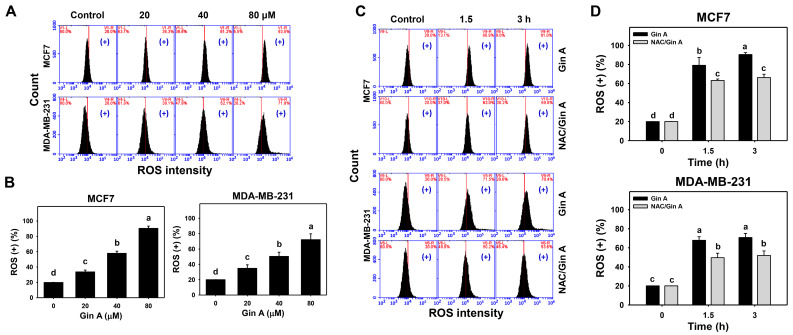
Impact of Gin A on ROS level of breast cancer cells. (**A**,**B**) ROS level. Cells were treated with 0 (0.1% DMSO; control), 20, 40, and 80 μM Gin A for 3 h. Subsequently, they were subjected to ROS flow cytometry. (+) indicates ROS (+) populations. (**C**,**D**) NAC reverted effects on ROS level. Cells were pretreated with NAC (10 mM) for 1 h and then post-treated with Gin A (control and 80 μM) for 0, 1.5, and 3 h, namely NAC/Gin A. Data, means ± SDs (*n* = 3). When the connecting letters are overlapping, it means their difference was significant for multiple comparisons (*p* < 0.05 to 0.0001). For the example of MCF7 cells (**D**), Gin A at 0, 1.5, and 3 h showing *d*, *b*, and *a* differs significantly. NAC/Gin A at 1.5 and 3 h showing *c* differs non-significantly.

The impact of Gin A on the ROS level of breast cancer cells (MCF7 and MDA-MB-231) was assessed using flow cytometric analysis (Figure 3A,C). Gin A dose-responsively promoted ROS levels in breast cancer cells (Figure 3B). Moreover, Gin A (80 μM) treatment at 1.5 and 3 h showed higher ROS levels in breast cancer cells than in the control (0 h) (Figure 3D).

By adding NAC, we assessed the impact of oxidative stress on the ROS level of Gin A in breast cancer cells. After NAC pretreatment, Gin A-induced ROS increments were partly reverted (Figure 3D).

### 3.4. Gin A Provoked MitoSOX Increment of Breast Cancer Cells

**Figure 4:**
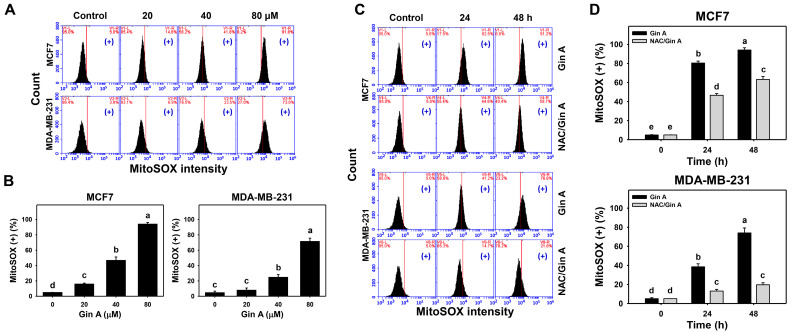
Impact of Gin A on MitoSOX level of breast cancer cells. (**A**,**B**) MitoSOX level. Cells were treated with 0 (0.1% DMSO; control), 20, 40, and 80 μM Gin A for 48 h. Subsequently, they were subjected to MitoSOX flow cytometry. (+) indicates MitoSOX (+) populations. (**C**,**D**) NAC reverted effects on MitoSOX level. Cells were pretreated with NAC (10 mM) for 1 h and then post-treated with Gin A (control and 80 μM) for 0, 24, and 48 h, namely NAC/Gin A. Data, means ± SDs (*n* = 3). When the connecting letters are overlapping, it means that their difference is significant for multiple comparisons (*p* < 0.05 to 0.0001). For the example of MCF7 cells (**D**), Gin A at 0, 24, and 48 h showing *e*, *b*, and *a* differs significantly. NAC/Gin A at 24 and 48 h showing *d* and *c* differs significantly, whereas Gin A and NAC/Gin A at 0 h showing the overlapped letter *e* differ non-significantly.

The impact of Gin A on the MitoSOX level of breast cancer cells (MCF7 and MDA-MB-231) was assessed by flow cytometric analysis (Figure 4A,C). Gin A dose-responsively promoted MitoSOX level in breast cancer cells (Figure 4B). Moreover, Gin A (80 μM) treatment at 24 and 48 h promoted MitoSOX level in breast cancer cells in a time-dependent manner (Figure 4D).

By adding NAC, we assessed the impact of oxidative stress on the MitoSOX level of Gin A in breast cancer cells. After NAC pretreatment, Gin A-induced MitoSOX increments were partly reverted (Figure 4D).

### 3.5. Gin A Provoked the Appearance of Senescence in Breast Cancer Cells

**Figure 5:**
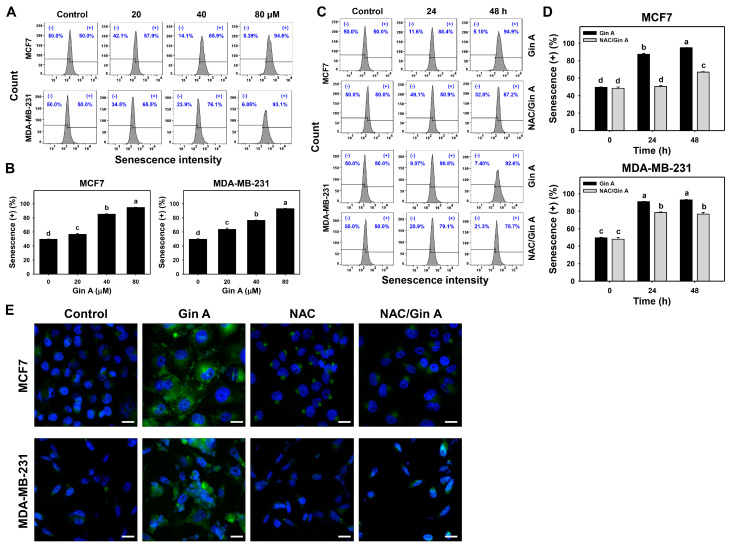
Impact of Gin A on senescence level and phenotype in breast cancer cells. (**A**,**B**) Senescence β-galactosidase level. Cells were treated with 0 (0.1% DMSO; control), 20, 40, and 80 μM Gin A for 48 h. Subsequently, they were subjected to senescence flow cytometry. (+) indicates senescence (+) populations. (**C**,**D**) NAC reverted effects on senescence level. Cells were pretreated with NAC (10 mM) for 1 h and then post-treated with Gin A (control and 80 μM) for 0, 24, and 48 h, namely NAC/Gin A. Data, means ± SDs (*n* = 3). When the connecting letters are overlapping, it means their difference was significant for multiple comparisons (*p* < 0.0001). For the example of MCF7 cells (**D**), Gin A at 0, 24, and 48 h showing *d*, *b*, and *a* differs significantly. NAC/Gin A at 24 and 48 h showing *d* and *c* differs significantly, whereas Gin A and NAC/Gin A at 0 h showing the overlapping letter *d* differ non-significantly. (**E**) Fluorescence analysis for β-galactosidase-detected senescence. Cells were stained by the senescence-detecting probe and counterstained with Hoechst 33342. Slides were photographed at 400× magnification. Scale bar = 20 μm.

Since fluorescence is more sensitive than visual light, the fluorescence-based β-galactosidase activity was measured using flow cytometry and fluorescence microscopy (Figure 5).

The impact of Gin A on senescence-inducing effects of breast cancer cells (MCF7 and MDA-MB-231) was assessed using flow cytometric analysis (Figure 5A,C). Gin A dose-responsively promoted senescence levels in breast cancer cells (Figure 5B). Moreover, Gin A (80 μM) treatment at 24 and 48 h showed higher senescence levels in breast cancer cells than control (0 h) (Figure 5D).

By adding NAC, we assessed the impact of oxidative stress on senescence level of Gin A in breast cancer cells. After NAC pretreatment, Gin A-induced senescence increments were partly reverted (Figure 5D).

Moreover, the impact of Gin A on senescence-inducing effects in breast cancer cells was also assessed by fluorescence microscopy (Figure 5E). β-galactosidase-detected senescence phenotype appeared in Gin A-treated breast cancer cells showing green fluorescence rather than in control. This Gin A-induced senescence phenotype was reduced by NAC pretreatment.

### 3.6. Gin A Provoked Senescence-Associated Gene Expressions of Breast Cancer Cells

**Figure 6:**
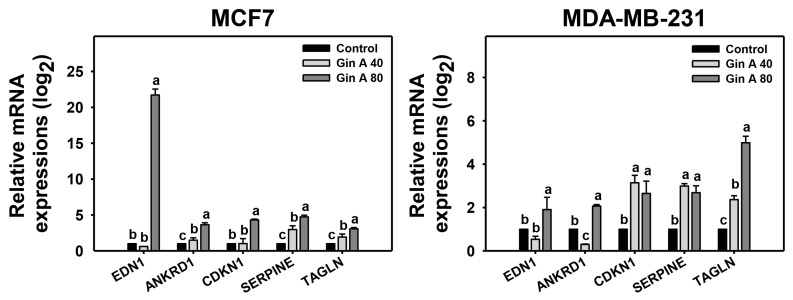
Impact of Gin A on senescence-associated gene expressions of breast cancer cells. Cells were treated with 0 (0.1% DMSO; control), 40, and 80 μM Gin A for 48 h, namely Gin A 40 and Gin A 80. Subsequently, the mRNA expressions of senescence-associated genes were examined by qRT-PCR. Data, means ± SDs (*n* = 3). When the connecting letters are overlapping, it means that their difference was significant for multiple comparisons (*p* < 0.05 to 0.0001). For the example of the *EDN1* gene in MCF7 and MDA-MB-231 cells, control and Gin A 40 showing *b* differ non-significantly. Gin A 80 showing *a* differs significantly from control and Gin A 40 showing *b* because they do not overlap with the same letter, i.e., *a* vs. *b*. For the example of the *TAGLN* gene in MCF7 and MDA-MB-231 cells, control, Gin A 40, and Gin A 80 showing *c*, *b*, and *a* differ significantly.

The impact of Gin A on senescence-inducing effects of breast cancer cells (MCF7 and MDA-MB-231) was also assessed using qRT-PCR analysis (Figure 6). Senescence-related genes [28,29] were detected, including *EDN1*, *ANKRD1*, *CDKN1A*, *SERPINE*, and *TAGLN*. Gin A at 80 μM showed higher mRNA levels of these senescence-related genes on breast cancer cells than control.

### 3.7. Gin A Provoked γH2AX Appearance of Breast Cancer Cells

**Figure 7:**
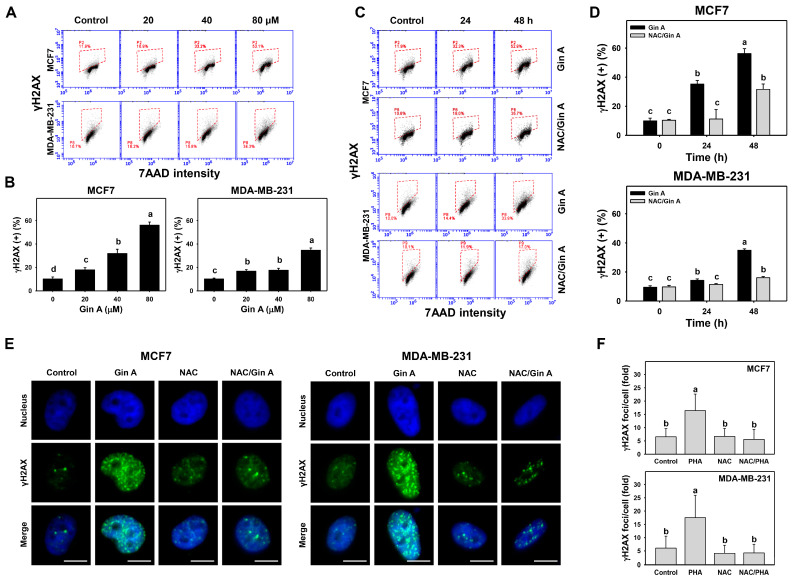
Impact of Gin A on γH2AX level and foci of breast cancer cells. (**A**,**B**) γH2AX level. Cells were treated with 0 (0.1% DMSO; control), 20, 40, and 80 μM Gin A for 48 h. Subsequently, they were subjected to γH2AX flow cytometry. Red boxes indicate γH2AX (+) populations. (**C**,**D**) NAC reverted effects on γH2AX level. Cells were pretreated with NAC (10 mM) for 1 h and then post-treated with Gin A (control and 80 μM) for 0, 24, and 48 h, namely NAC/Gin A. Data, means ± SDs (*n* = 3). When the connecting letters are overlapping, this means that their difference was significant for multiple comparisons (*p* < 0.0001). For the example of MCF7 cells (**D**), Gin A at 0, 24, and 48 h showing *c*, *b*, and *a* differs significantly. NAC/Gin A at 24 and 48 h showing *c* and *b* differs significantly, whereas Gin A and NAC/Gin A at 0 and 24 h showing the overlapping letter *c* differ non-significantly. (**E**,**F**) Fluorescence analysis for γH2AX foci. Cells were stained by the senescence-detecting probe and counterstained with Hoechst 33342. Slides were photographed at 400× magnification. Scale bar = 10 μm. DNA damage was counted as γH2AX foci per cell. Data, means ± SDs (*n* = 20 cells).

The impact of Gin A on the γH2AX level in breast cancer cells (MCF7 and MDA-MB-231) was assessed by flow cytometric analysis (Figure 7A,C). Gin A dose-responsively promoted γH2AX levels in breast cancer cells (Figure 7B). Moreover, in a time-dependent manner, Gin A (80 μM) treatment at 24 and 48 h elevated γH2AX levels in breast cancer cells (Figure 7D).

By adding NAC, we assessed the impact of oxidative stress on the γH2AX level of Gin A in breast cancer cells. After NAC pretreatment, Gin A-induced γH2AX increments were partly reverted (Figure 7D).

Moreover, the impact of Gin A on γH2AX foci in breast cancer cells was assessed using fluorescence microscopy (Figure 7E). γH2AX foci appeared in Gin A-treated breast cancer cells showing green fluorescence spots rather than in control (Figure 7F). These Gin A-induced γH2AX foci increments were reduced by NAC pretreatment.

### 3.8. Gin A Provoked 8-OHdG Increment of Breast Cancer Cells

**Figure 8:**
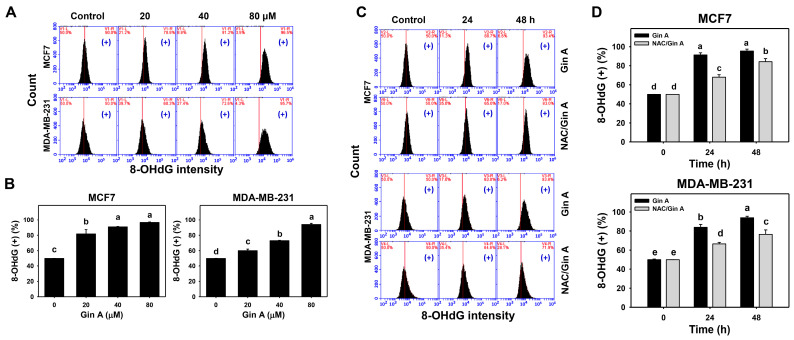
Impact of Gin A on 8-OHdG level in breast cancer cells. (**A**,**B**) 8-OHdG level. Cells were treated with 0 (0.1% DMSO; control), 20, 40, and 80 μM Gin A for 48 h. Subsequently, they were subjected to 8-OHdG flow cytometry. (+) indicates 8-OHdG (+) populations. (**C**,**D**) NAC reverted effects on the 8-OhdG level. Cells were pretreated with NAC (10 mM) for 1 h and then post-treated with Gin A (control and 80 μM) for 0, 24, and 48 h, namely NAC/Gin A. Data, means ± SDs (*n* = 3). When the connecting letters are overlapping, their difference was significant for multiple comparisons (*p* < 0.05 to 0.0001). For the example of MDA-MB-231 cells (**D**), Gin A at 0, 24, and 48 h showing *e*, *b*, and *a* differs significantly. NAC/Gin A at 0, 24, and 48 h showing *e*, *d*, and *c* differs significantly, whereas Gin A and NAC/Gin A at 0 h showing the overlapped letter *e* differ non-significantly.

The impact of Gin A on the 8-OHdG level of breast cancer cells (MCF7 and MDA-MB-231) was assessed using flow cytometric analysis (Figure 8A,C). Gin A dose-responsively promoted 8-OHdG level in breast cancer cells (Figure 8B). Moreover, Gin A (80 μM) treatment at 24 and 48 h elevated 8-OHdG levels in breast cancer cells more than in the control (0 h) (Figure 8D).

Using adding NAC, we assessed the impact of oxidative stress on the 8-OHdG level of Gin A in breast cancer cells. After NAC pretreatment, Gin A-induced 8-OHdG increments were partly reverted (Figure 8D).

## 4. Discussion

The present study reported that Gin A showed antiproliferation and senescence properties in breast cancer cells. The relationship between senescence-associated changes and DNA damages connecting to oxidative stress was discussed.

The present study reported an IC_50_ of 50.41, 42.67, and 56.29 μM for Gin A in SKBR3, MCF7, and MDA-MB-231 cells based on the 48 h ATP assay (Figure 1). In 48 h MTS assay, Gin A exhibited an IC_50_ value of 48.91, 61.40, and 76.12 μM in breast cancer cells (SKBR3, MCF 7, and MDA-MB-231). The anticancer effect of Gin A was reported in liver cancer Sk-Hep-1 cells; i.e., the IC_50_ value was 27.5 μM at 24 h trypan blue assay [11]. Gin A at 50 μM showed subG1 accumulation, cytochrome C release, mitochondrial membrane potential depletion, and reactive oxygen species (ROS) induction in liver cancer Sk-Hep-1 cells [11]. The drug’s safety to normal cell lines has been reported in epithelial MDCK (Madin–Darby canine kidney) [37], showing non-cytotoxicity at 25 μM and 80% viability at 50 μM based on 24 h trypan blue assay [11]. For comparison, breast cancer (SKBR3 and MCF7) showed IC_50_ values of 12.37 and 34.83 µM for cisplatin in 48 h MTS assay [38]. Breast cancer cells (SKBR3, MCF7, and MDA-MB-231) showed IC_50_ values of 4.9, 17.9, and 26.9 µM for cisplatin in 48 h ATP study [39]. MCF7 cells showed IC_50_ values of 59.12, 28.10, 23.00, 52.18, 12.10, and 359.47 µM for caffeic acid, caffeic acid phenethyl ester [40], thymoquinone [41], berberine [42], aripiprazole [43], and thymol [44] in 48 h XTT or MTT studies. Therefore, the drug sensitivity of Gin A is shown to be less than that of cisplatin but shows similar results compared to other biomolecules, as described.

Several bioactive compounds, such as gingerols, shogaols, gingediols, zingerone, dehydrozingerone, gingerinone, and diarylheptanoids, have been isolated from ginger extracts [8,9,10]. Some of these ginger derivatives were reported to exhibit oxidative stress-inducing ability. For example, (6)-Gingerol can induce ROS generation and apoptosis in gastric cancer cells [45]. 6-Shogaol promoted ROS and endoplasmic reticulum stress so as to trigger apoptosis in endometrial cancer cells [46]. Gingediols caused ROS-dependent apoptosis in colon cancer cells [47]. Similarly, Gin A caused oxidative stress by inducing ROS (Figure 3) and MitoSOX (Figure 4) in a dose- and time-dependent manner. These reports suggest that ginger derivatives have a potential ROS-modulating function.

Oxidative stress causes oxidative DNA damage [48,49]. Moreover, oxidative stress can cause DNA damage to initiate senescence [50]. DNA damage exhibits senescence-inducing effects on cancer cells in vitro and in vivo [51]. For example, oncogenic RAS upregulating mitochondrial mass and ROS can induce DNA damage and senescence [52]. Similarly, Gin A upregulated γH2AX level and foci as detected by flow cytometry and fluorescence microscopy (Figure 7). Gin A also provoked oxidative DNA damage as detected by 8-OHdG flow cytometry (Figure 8).

Ginger extract can induce senescence in lung cancer cells [53] or protect the senescence of myoblasts [54]. Accordingly, the senescence responses of ginger extract still need further investigation. Although ginger derivative 6-Shogaol promoted apoptosis in endometrial cancer cells [46], it suppressed apoptosis and senescence in human dermal fibroblasts [55]. Conversely, Gin A induced β-galactosidase activity in breast cancer cells as detected by flow cytometry and fluorescence microscopy (Figure 5), suggesting that Gin A induced senescence in breast cancer cells. Therefore, different bioactive compounds of ginger may exhibit different responses to senescence. The nature of cell types also needs to be considered for the senescence response to ginger derivatives.

Several senescence genes (*EDN1*, *ANKRD1*, *CDKN1A*, *SERPINE*, and *TAGLN*) were reported [28,29]. These genes were upregulated at 48 h Gin A treatment for breast cancer cells (MCF7 and MDA-MB-231) (Figure 6). The expression status for these senescence genes was different for these cell lines. For example, *EDN1* mRNA was highly expressed among these senescence-associated genes in MCF7 cells but only slightly expressed in MDA-MB-231 cells. In contrast, *TAGLN* mRNA was highly expressed among these senescence-associated genes in MDA-MB-231 cells but expressed only somewhat in MCF7 cells. These results indicate that Gin A differentially regulates different senescence-associated genes in different breast cancer cell lines.

NAC pretreatment could revert the changes for proliferation, cell cycle progression, oxidative stress-associated modulations, senescence phenotypes, and DNA damages in breast cancer cells (Figure 1, Figure 2, Figure 3, Figure 4, Figure 5, Figure 7 and Figure 8). These results indicate that oxidative stress plays a central role in the antiproliferation and senescence effects of Gin A in breast cancer cells.

## 5. Conclusions

Ginger is a popular spice that is famous for several of its bioactive compounds. Gin A is a novel compound isolated from ginger. We report for the first time that Gin A induced antiproliferation and senescence responses acting on breast cancer cells. Senescence-associated genes were also upregulated by Gin A. Several experiments confirmed that Gin A generated oxidative stress and DNA damages to breast cancer cells. These responses to Gin A in breast cancer cells were confirmed to be ROS-dependent using NAC pretreatment. Therefore, Gin A is an antiproliferation- and senescence-inducing natural product against breast cancer cells involving oxidative-stress-associated responses.

## Figures and Tables

**Table 1:** Primer information for senescence-related genes.

Genes	Forward Primers (5′→3′)	Reverse Primers (5′→3′)	Length
*EDN1*	CAGCAGTCTTAGGCGCTGAG	ACTCTTTATCCATCAGGGACGAG	126 bp
*ANKRD1*	AGTAGAGGAACTGGTCACTGG	TGGGCTAGAAGTGTCTTCAGAT	138 bp
*CDKN1A*	GACACCACTGGAGGGTGACT	CAGGTCCACATGGTCTTCCT	172 bp
*SERPINE1*	GTGTTTCAGCAGGTGGCGC	CCGGAACAGCCTGAAGAAGTG	300 bp
*TAGLN*	TGGCGTGATTCTGAGCAAG	ACTGCCAAGCTGCCCAA	240 bp
*GAPDH*	CCTCAACTACATGGTTTACATGTTCC	CAAATGAGCCCCAGCCTTCT	220 bp

## Data Availability

Data are contained within the article.

## References

[1] Sung H., Ferlay J., Siegel R.L., Laversanne M., Soerjomataram I., Jemal A., Bray F. (2021). Global Cancer Statistics 2020: GLO-BOCAN estimates of incidence and mortality worldwide for 36 cancers in 185 countries. CA Cancer J. Clin..

[2] Holliday D.L., Speirs V. (2011). Choosing the right cell line for breast cancer research. Breast Cancer Res..

[3] Lehmann B.D., Pietenpol J.A. (2014). Identification and use of biomarkers in treatment strategies for triple-negative breast cancer subtypes. J. Pathol..

[4] Burstein M.D., Tsimelzon A., Poage G.M., Covington K.R., Contreras A., Fuqua S.A., Savage M.I., Osborne C.K., Hilsenbeck S.G., Chang J.C. (2015). Comprehensive Genomic Analysis Identifies Novel Subtypes and Targets of Triple-Negative Breast Cancer. Clin. Cancer Res..

[5] Pagano E., Souto E.B., Durazzo A., Sharifi-Rad J., Lucarini M., Souto S.B., Salehi B., Zam W., Montanaro V., Lucariello G. (2021). Ginger (*Zingiber officinale Roscoe*) as a nutraceutical: Focus on the metabolic, analgesic, and antiinflammatory effects. Phytother. Res..

[6] Cheema H.S., Singh M.P. (2021). The Use of Medicinal Plants in Digestive System Related Disorders—A Systematic Review. J. Ayurvedic Herb. Med..

[7] Zhang M., Zhao R., Wang D., Wang L., Zhang Q., Wei S., Lu F., Peng W., Wu C. (2021). Ginger (*Zingiber officinale Roscoe*) and its bioactive components are potential resources for health beneficial agents. Phytother. Res..

[8] Kubra I.R., Rao L.J.M. (2012). An Impression on Current Developments in the Technology, Chemistry, and Biological Activities of Ginger (*Zingiber officinale Roscoe*). Crit. Rev. Food Sci. Nutr..

[9] Liu C.-M., Kao C.-L., Tseng Y.-T., Lo Y.-C., Chen C.-Y. (2017). Ginger Phytochemicals Inhibit Cell Growth and Modulate Drug Resistance Factors in Docetaxel Resistant Prostate Cancer Cell. Molecules.

[10] Oboh G., Akinyemi A.J., Ademiluyi A.O. (2012). Antioxidant and inhibitory effect of red ginger (*Zingiber officinale var. Rubra*) and white ginger (*Zingiber officinale Roscoe*) on Fe2+ induced lipid peroxidation in rat brain in vitro. Exp. Toxicol. Pathol..

[11] Mann T.F., Chen C.Y., Chern C.L., Liou S.S., Yiin S.J. (2015). Gingernone A-induced apoptosis in SK-Hep-1 cells is mediated via increased reactive oxygen species (ROS) production and the mitochondria-associated apoptotic mechanisms. Tajen J..

[12] Wang B., Kohli J., Demaria M. (2020). Senescent Cells in Cancer Therapy: Friends or Foes?. Trends Cancer.

[13] Prasanna P.G., Citrin D.E., Hildesheim J., Ahmed M.M., Venkatachalam S., Riscuta G., Xi D., Zheng G., van Deursen J.V., Goronzy J. (2021). Therapy-Induced Senescence: Opportunities to Improve Anticancer Therapy. JNCI J. Natl. Cancer Inst..

[14] Pole A., Dimri M., Dimri G.P. (2016). Oxidative stress, cellular senescence and ageing. AIMS Mol. Sci..

[15] Li Y., Li W., Ying Z., Tian H., Zhu X., Li J., Li M. (2014). Metastatic Heterogeneity of Breast Cancer Cells Is Associated with Expression of a Heterogeneous TGFβ-Activating miR424–503 Gene Cluster. Cancer Res..

[16] Gómez-Cuadrado L., Tracey N., Ma R., Qian B., Brunton V.G. (2017). Mouse models of metastasis: Progress and prospects. Dis. Model. Mech..

[17] Huang H.W., Tang J.Y., Ou-Yang F., Wang H.R., Guan P.Y., Huang C.Y., Chen C.Y., Hou M.F., Sheu J.H., Chang H.W. (2018). Sinularin selectively kills breast cancer cells showing G2/M arrest, apoptosis, and oxidative DNA damage. Molecules.

[18] Chan W.H., Shiao N.H., Lu P.Z. (2006). CdSe quantum dots induce apoptosis in human neuroblastoma cells via mitochondri-al-dependent pathways and inhibition of survival signals. Toxicol. Lett..

[19] Hung J.-H., Chen C.-Y., Omar H.A., Huang K.-Y., Tsao C.-C., Chiu C.-C., Chen Y.-L., Chen P.-H., Teng Y.-N. (2015). Reactive oxygen species mediate Terbufos-induced apoptosis in mouse testicular cell lines via the modulation of cell cycle and pro-apoptotic proteins. Environ. Toxicol..

[20] Huang C.-H., Yeh J.-M., Chan W.-H. (2018). Hazardous impacts of silver nanoparticles on mouse oocyte maturation and fertilization and fetal development through induction of apoptotic processes. Environ. Toxicol..

[21] Wang T.-S., Lin C.-P., Chen Y.-P., Chao M.-R., Li C.-C., Liu K.-L. (2018). CYP450-mediated mitochondrial ROS production involved in arecoline N -oxide-induced oxidative damage in liver cell lines. Environ. Toxicol..

[22] Chen C.Y., Yen C.Y., Wang H.R., Yang H.P., Tang J.Y., Huang H.W., Hsu S.H., Chang H.W. (2016). Tenuifolide B from Cin-namomum tenuifolium stem selectively inhibits proliferation of oral cancer cells via apoptosis, ROS generation, mitochondrial depolarization, and DNA damage. Toxins.

[23] Chang H.-W., Li R.-N., Wang H.-R., Liu J.-R., Tang J.-Y., Huang H.-W., Chan Y.-H., Yen C.-Y. (2017). Withaferin A Induces Oxidative Stress-Mediated Apoptosis and DNA Damage in Oral Cancer Cells. Front. Physiol..

[24] Chiu C.C., Chang H.W., Chuang D.W., Chang F.R., Chang Y.C., Cheng Y.S., Tsai M.T., Chen W.Y., Lee S.S., Wang C.K. (2009). Fern plant-derived protoapigenone leads to DNA damage, apoptosis, and G(2)/M arrest in lung cancer cell line H1299. DNA Cell Biol..

[25] Tang J.Y., Wu C.Y., Shu C.W., Wang S.C., Chang M.Y., Chang H.W. (2018). A novel sulfonyl chromen-4-ones (CHW09) prefer-entially kills oral cancer cells showing apoptosis, oxidative stress, and DNA damage. Environ. Toxicol..

[26] Beltran M., Tavares M., Justin N., Khandelwal G., Ambrose J., Foster B.M., Worlock K.B., Tvardovskiy A., Kunzelmann S., Herrero J. (2019). G-tract RNA removes Polycomb repressive complex 2 from genes. Nat. Struct. Mol. Biol..

[27] Yen C.-Y., Huang C.-Y., Hou M.-F., Yang Y.-H., Chang C.-H., Huang H.-W., Chen C.-H., Chang H.-W. (2012). Evaluating the performance of fibronectin 1 (FN1), integrin α4β1 (ITGA4), syndecan-2 (SDC2), and glycoprotein CD44 as the potential biomarkers of oral squamous cell carcinoma (OSCC). Biomarkers.

[28] Hooten N.N., Evans M.K. (2017). Techniques to Induce and Quantify Cellular Senescence. J. Vis. Exp..

[29] Chen J.Y.-F., Hwang C.-C., Chen W.-Y., Lee J.-C., Fu T.-F., Fang K., Chu Y.-C., Huang Y.-L., Lin J.-C., Tsai W.-H. (2010). Additive effects of C2-ceramide on paclitaxel-induced premature senescence of human lung cancer cells. Life Sci..

[30] Livak K.J., Schmittgen T.D. (2001). Analysis of relative gene expression data using real-time quantitative PCR and the 2^−ΔΔC_T_^ Method. Methods.

[31] Fujii Y., Yoshihashi K., Suzuki H., Tsutsumi S., Mutoh H., Maeda S., Yamagata Y., Seto Y., Aburatani H., Hatakeyama M. (2012). CDX1 confers intestinal phenotype on gastric epithelial cells via induction of stemness-associated reprogramming factors SALL4 and KLF5. Proc. Natl. Acad. Sci.USA.

[32] Laddha N.C., Dwivedi M., Mansuri M.S., Singh M., Patel H.H., Agarwal N., Shah A.M., Begum R. (2014). Association of Neuropeptide Y (NPY), Interleukin-1B (IL1B) Genetic Variants and Correlation of IL1B Transcript Levels with Vitiligo Susceptibility. PLoS ONE.

[33] Tang J.Y., Huang H.W., Wang H.R., Chan Y.C., Haung J.W., Shu C.W., Wu Y.C., Chang H.W. (2018). 4beta-Hydroxywithanolide E selectively induces oxidative DNA damage for selective killing of oral cancer cells. Environ. Toxicol..

[34] Bahreyni-Toossi M.T., Azimian H., Aghaee-Bakhtiari S.H., Mahmoudi M., Sadat-Darbandi M., Zafari N. (2021). Radiation-induced DNA damage and altered expression of p21, cyclin D1 and Mre11 genes in human fibroblast cell lines with different radio-sensitivity. Mutat. Res..

[35] Omari Shekaftik S., Nasirzadeh N. (2021). 8-Hydroxy-2′-deoxyguanosine (8-OHdG) as a biomarker of oxidative DNA damage in-duced by occupational exposure to nanomaterials: A systematic review. Nanotoxicology.

[36] Valavanidis A., Vlachogianni T., Fiotakis C. (2009). 8-hydroxy-2′ -deoxyguanosine (8-OHdG): A Critical Biomarker of Oxidative Stress and Carcinogenesis. J. Environ. Sci. Health Part C.

[37] Simons K., Virta H., Celis J.E. (2006). Growing Madin-Darby Canine Kidney Cells for Studying Epithelial Cell Biology. Cell Biology.

[38] Ou-Yang F., Tsai I.H., Tang J.Y., Yen C.Y., Cheng Y.B., Farooqi A.A., Chen S.R., Yu S.Y., Kao J.K., Chang H.W. (2019). Anti-proliferation for breast cancer cells by ethyl acetate extract of *Nepenthes thorellii* × *(ventricosa* × *maxima*). Int. J. Mol. Sci..

[39] Yu T.J., Tang J.Y., Lin L.C., Lien W.J., Cheng Y.B., Chang F.R., Ou-Yang F., Chang H.W. (2020). Withanolide C inhibits prolifer-ation of breast cancer cells via oxidative stress-mediated apoptosis and DNA damage. Antioxidants.

[40] Kabała-Dzik A., Rzepecka-Stojko A., Kubina R., Wojtyczka R.D., Buszman E., Stojko J. (2018). Caffeic Acid Versus Caffeic Acid Phenethyl Ester in the Treatment of Breast Cancer MCF-7 Cells: Migration Rate Inhibition. Integr. Cancer Ther..

[41] Hamid S.S., Motaghed M., Al-Hassan F.M. (2013). Cellular responses with thymoquinone treatment in human breast cancer cell line MCF-7. Pharmacogn. Res..

[42] Zhao Y., Jing Z., Li Y., Mao W. (2016). Berberine in combination with cisplatin suppresses breast cancer cell growth through in-duction of DNA breaks and caspase-3-dependent apoptosis. Oncol. Rep..

[43] Badran A., Tul-Wahab A., Zafar H., Mohammad N., Imad R., Ashfaq Khan M., Baydoun E., Choudhary M.I. (2020). Antipsy-chotics drug aripiprazole as a lead against breast cancer cell line (MCF-7) in vitro. PLoS ONE.

[44] Duarte D., Vale N. (2020). New Trends for Antimalarial Drugs: Synergism between Antineoplastics and Antimalarials on Breast Cancer Cells. Biomolecules.

[45] Mansingh D.P., Sunanda O.J., Sali V.K., Vasanthi H.R. (2018). [6]-Gingerol-induced cell cycle arrest, reactive oxygen species generation, and disruption of mitochondrial membrane potential are associated with apoptosis in human gastric cancer (AGS) cells. J. Biochem. Mol. Toxicol..

[46] Ma R.-H., Ni Z.-J., Zhang F., Zhang Y.-Y., Liu M.-M., Thakur K., Zhang J.-G., Wang S.Y., Wei Z.-J. (2020). 6-Shogaol mediated ROS production and apoptosis via endoplasmic reticulum and mitochondrial pathways in human endometrial carcinoma Ishikawa cells. J. Funct. Foods.

[47] Su P., Veeraraghavan V.P., Krishna Mohan S., Lu W. (2019). A ginger derivative, zingerone-a phenolic compound-induces ROS-mediated apoptosis in colon cancer cells (HCT-116). J. Biochem. Mol. Toxicol..

[48] Bohr V.A., Dianov G.L. (1999). Oxidative DNA damage processing in nuclear and mitochondrial DNA. Biochimie.

[49] Salehi F., Behboudi H., Kavoosi G., Ardestani S.K. (2018). Oxidative DNA damage induced by ROS-modulating agents with the ability to target DNA: A comparison of the biological characteristics of citrus pectin and apple pectin. Sci. Rep..

[50] Zhu M.-J., Wang X., Shi L., Liang L.-Y., Wang Y. (2018). Senescence, oxidative stress and mitochondria dysfunction. Cancer Med. Res. Innov..

[51] Te Poele R.H., Okorokov A.L., Jardine L., Cummings J., Joel S.P. (2002). DNA damage is able to induce senescence in tumor cells in vitro and in vivo. Cancer Res..

[52] Moiseeva O., Bourdeau V., Roux A., Deschenes-Simard X., Ferbeyre G. (2009). Mitochondrial dysfunction contributes to onco-gene-induced senescence. Mol. Cell. Biol..

[53] Kaewtunjai N., Wongpoomchai R., Imsumran A., Pompimon W., Athipornchai A., Suksamrarn A., Lee T.R., Tuntiwechapikul W. (2018). Ginger Extract Promotes Telomere Shortening and Cellular Senescence in A549 Lung Cancer Cells. ACS Omega.

[54] Mohd Sahardi N.F.N., Jaafar F., Mad Nordin M.F., Makpol S. (2020). Zingiber officinale Roscoe prevents cellular senescence of my-oblasts in culture and promotes muscle regeneration. Evid. Based Complement. Altern. Med..

[55] Han H.S., Kim K.B., Jung J.H., An I.S., Kim Y.-J., An S. (2018). Anti-apoptotic, antioxidant and anti-aging effects of 6-shogaol on human dermal fibroblasts. Biomed. Dermatol..

